# Cholesterol polyps in the distal common bile duct: a case report

**DOI:** 10.1097/MD.0000000000005374

**Published:** 2016-11-11

**Authors:** Rui Tang, Wen-ping Zhao, Yan-ning Zhang, Xuan Tong, Jian-ping Zeng

**Affiliations:** Department of Hepatopancreatobiliary Surgery, Beijing Tsinghua Changgung Hospital, Tsinghua University Medical Center, Beijing, China.

**Keywords:** choledochoscopic examination, cholesterol polyp of the common bile duct, cholesterosis, foam cell, ursodesoxycholic acid

## Abstract

**Rationale:**

Cholesterol polyps are rare in the common bile duct and difficult to diagnose.

**Patient concerns:**

The small polypoid lesions often go undetected when using routine imaging methods, such as ultrasonography.

**Diagnoses:**

We treated a patient with cholesterol polyps in the common bile duct. After failing to detect choleliths using ultrasonography, magnetic resonance cholangiopancreatography revealed mild dilation of the common bile duct. Choledochoscopy was performed during laparoscopic cholecystectomy, which revealed yellowish-white polyps circumferentially distributed across the luminal surface of the distal common bile duct. Histological examination of biopsy specimens indicated cholesterol polyps with characteristic foamy cells.

**Interventions:**

The patient was treated with ursodeoxycholic acid, and the number of polyps was found to have been reduced at the 6-week follow-up based on T-tube choledochoscopic examination.

**Outcomes:**

Recovery was unremarkable, and the ursodeoxycholic acid treatment was discontinued at the 6-month follow-up.

**Lessons subsections:**

Our findings suggest that this rare condition can be treated pharmacologically to avoid potential postsurgical complications following resection of the distal common bile duct.

## 1 Introduction

Cholesterol polyps make up approximately 50% of all polypoid lesions in the gallbladder, but are rare in the common bile duct (CBD).^[1,2]^ With an average diameter of 3 to 10 mm,^[3]^ the small size of cholesterol polyps confounds diagnosis by ultrasonography and other routine imaging methods. The development of cholesterol polyps is associated with cholangiocarcinoma.^[4]^ Therefore, choledochoscopic biopsy is essential for the histopathological diagnosis of cholesterol polyps to rule out malignancy.^[1]^ We encountered a patient with cholesterol polyps in the distal CBD. Choledochoscopic examination was crucial for the diagnosis of this rare condition.

## 2 Case report

Our report of this case was approved by the Medical Ethics Committee of our institution, and signed, informed consent was obtained from the patient. A 53-year-old woman presented to our hospital with upper right quadrant pain. She was not jaundice, and had no fever. Her medical history was unremarkable, with no recent diarrhea, pruritus, dark urine, or weight loss reported. Ultrasonography showed thickening of the gallbladder wall and no obvious dilation or filling defects in the extrahepatic biliary tract. However, magnetic resonance cholangiopancreatography (MRCP) revealed mild dilation of the CBD, which suggested a diagnosis of cholecystitis with cholelithiasis, but choledocholithiasis could not be excluded.

Treatment with laparoscopic cholecystectomy and intraoperative choledochoscopic examination was chosen. The resected gallbladder contained a large amount of sand-like cholelithic material, with particles approximately 1 to 2 mm in diameter. The anterior side of the midpoint of the CBD was incised for intraoperative choledochoscopic examination of the lumen and its contents. Extensive minimal papillary projections were observed in the distal CBD that were yellowish-white in color, but no choleliths were present. Polyp biopsy was performed, and a T-tube biliary drainage line was installed. Histological examination of the biopsy specimen revealed foamy cell aggregation in the lamina propria, indicating cholesterol polyps.

**Figure 1:**
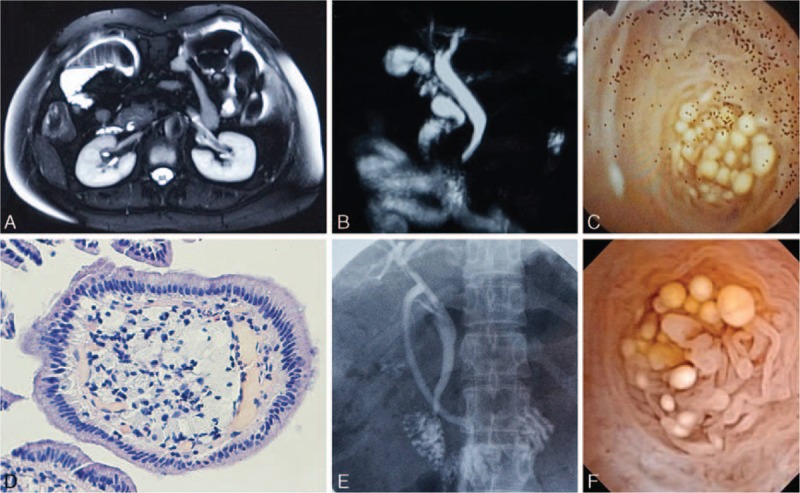
Imaging and histological data. Preoperative MRCP images in the (A) transverse plane and (B) coronal plane (reconstructed) showed patent confluence of the pancreatic duct and CBD, with mild dilation of the latter, but no obvious filling defect. C, Intraoperative choledochoscopy revealed small, yellowish-white polypoid lesions in the distal CBD. D, Histological examination of biopsy specimens identified clusters of foamy cells within the lamina propria. After 6 weeks of treatment with 200 mg UDCA orally 2 times per day, (E) TTC showed no obvious filling defect in the CBD, and (F) choledochoscopy showed substantially fewer polyps. CBD = common bile duct, MRCP = magnetic resonance cholangiopancreatography, TTC = T-tube cholangiography, UDCA = ursodesoxycholic acid.

The small polyps were distributed across the entire circumference of the luminal surface of the distal CBD, and extended deep into the duodenal papilla. In our experience, resection of this region of the distal CBD might cause stenosis or sphincter dysfunction. Therefore, pharmacological treatment was chosen to reduce the size and number of polyps. The patient received 200 mg ursodesoxycholic acid (UDCA) twice daily per os. At the 6-week follow-up examination T-tube cholangiography (TTC) showed no obvious filling defect, and choledochoscopic examination revealed fewer polyps than were observed previously (Fig. 1), at which time the biliary drainage line was removed. Recovery was unremarkable, and UDCA treatment was discontinued at the 6-month follow-up examination.

## 3 Discussion

Cholesterol polyps in the CBD were first reported by Fock^[5]^ in 1958. To date, less than 40 such cases have been reported in the literature,^[4–8]^ and our case represents the first report of CBD cholesterol polyps in a patient in China. These small lesions are often undetectable using ultrasonography, computed tomography, or MRCP, whereas endoscopic retrograde cholangiopancreatography or percutaneous transhepatic cholangiography can be helpful for diagnosis in certain patients.^[6]^ In our patient, cholesterol polyps in the distal CBD were not detected by MRCP, which revealed only mild dilation, and choledochoscopy was performed for direct visualization to avoid misdiagnosis.

The exact etiology of cholesterol polyps in the gallbladder and biliary ducts is largely unclear, but it likely involves the crystallization of cholesterol in the lumen mucosa due to an elevated concentration of cholesterol in the bile.^[9]^ The condition occurs more frequently in women than in men. In most patients, cholesterol polyps occur in the distal CBD, and are accompanied by cholelithiasis. However, it has also been reported in patients with congenital choledochal cyst or anomalous bifurcation of the pancreaticobiliary ducts.^[10]^ Cholesterol polyps are associated with the tumorigenesis of cholangiocarcinoma, in which histological analysis of the polyps has revealed distinct differences between the benign and malignant conditions.^[4]^ Therefore, direct visual examination and histopathological analysis is essential to avoid misdiagnosis.^[8]^

Although cholesterol polyps can be resected from the distal CBD during choledochoscopy using biopsy forceps or spoons, the risk of biliary obstruction due to this condition is, in our experience, very low. In most cases, we believe that radical resection can result in unnecessary bile duct injury, as well as duodenal papillary dysfunction for cases in which the polyps extended into the papilla. The dissolution of cholesterol crystals can be achieved by the treatment with UDCA orally.^[11]^ Our findings warrant future studies of UDCA treatment as an alternative to CBD resection for cholesterol polyps. However, we recommend choledochoscopic examination to confirm the therapeutic effect of UDCA treatment. To the best of our knowledge, this is the first report of UDCA therapy for CBD cholesterol polyps.

## Acknowledgments

The authors thank Dr Yi-Ting Meng and Dr Jing-Jing Yao in the Pathology Department at Beijing Tsinghua Changgung Hospital for their assistance in determining the diagnosis.
